# Patterns of Perfusion of Free ALT Flaps vs. Propeller Flaps of the Lower Extremity: A Comparative Study with the Use of LDSP †

**DOI:** 10.3390/healthcare13192441

**Published:** 2025-09-26

**Authors:** Silvia Bernuth, Tobias Niederegger, Gabriel Hundeshagen, Konrad Fuchs, Rainer H. Meffert, Rafael G. Jakubietz

**Affiliations:** 1 Division of Plastic and Reconstructive Surgery, Clinic of Trauma, Hand, Plastic and Reconstructive Surgery, University Hospital Wuerzburg, 97080 Wuerzburg, Germany; 2 Division of Plastic, Reconstructive and Aesthetic Surgery, Clinic of Oral and Maxillofacial Surgery/Plastic Surgery, University Hospital Greifswald, 17475 Greifswald, Germany; 3 Medical Faculty, University of Heidelberg, 69120 Heidelberg, Germany; 4 Department of Hand, Plastic and Reconstructive Surgery, Burn Center, BG Trauma Center Ludwigshafen, University of Heidelberg, 67071 Ludwigshafen, Germany; 5 Clinic of Trauma, Hand, Plastic and Reconstructive Surgery, University Hospital Wuerzburg, 97080 Wuerzburg, Germany

**Keywords:** soft tissue reconstruction, perforator flap, lower extremity, perfusion

## Abstract

**Introduction:** Perforator-based fasciocutaneous flaps are particularly suitable for soft tissue reconstruction of the lower extremities. The most commonly used flap is the microvascular Anterior Lateral Thigh Flap (ALT). Pedicled propeller-type flaps are less frequently utilized due to higher complication rates. The aim of this study was to compare postoperative perfusion patterns of these fundamentally different flaps to increase their basic understanding. **Methods:** A retrospective data analysis was performed (2017–2022), including patients who underwent flap reconstruction of the lower extremity either with a perforator-based pedicled Propeller flap or free tissue transfer with an ALT flap. Only patients with documented postoperative perfusion monitoring of the flap using the laser Doppler spectrophotometry system (LDSP) were included. Demographic data, comorbidities and perioperative data as well as perfusion profiles given by the LDSP were analyzed. **Results:** Seven patients who received a propeller flap and 18 who received a free ALT were identified. Defects were most often due to trauma (Propeller flap *n* = 5; 71.1%; ALT *n* = 7; 38.9%) and chronic wounds (Propeller flap *n* = 1; 14.3%; ALT *n* = 5; 27.8%). The most common complication was prolonged wound healing (Propeller flap *n* = 3; 42.9%; ALT *n* = 8; 44.4%). In cases with postoperative surgical complications, a distinctly delayed recovery in perfusion of propeller flaps was seen during the first 72 h after surgery. **Conclusions:** Propeller and ALT flaps exhibit distinct perfusion patterns, with Propeller flaps showing a congestion-prone profile (elevated rHB, delayed hyperperfusion) and ALT flaps an inflow-dependent, ischemia-driven profile (lower rHB and SO_2_).

## 1. Introduction

Due to its thin, inelastic soft tissue coverage, the lower extremity is often associated with complex soft tissue defects after trauma or tumor resection. The advent of microvascular tissue transfer has significantly improved outcomes of lower extremity reconstruction. Perforator-based fasciocutaneous flaps are particularly suitable for soft tissue reconstruction of the lower extremity as they transfer viable and pliable tissue, including a well-vascularized fascia [1,2].

The microvascular Anterior Lateral Thigh Flap (ALT) was introduced by Song, Chen and Song [1] and became a workhorse flap of reconstructive microsurgery. This perforator flap is characterized by its large size, a reliably long pedicle, and a large vessel diameter [2,3,4,5,6]. Technical refinements such as thinning [5,7], inclusion of muscles [5,8] and sensory nerves [5,7], or the use as a flow-through flap [5,7] have further expanded its applications, making it an ideal flap for numerous aspects of complex wounds and revision procedures [5,7,9].

The advent of perforator surgery also rekindled an interest in local perforator-based alternatives, such as the propeller flap. The vascular pedicle serves as the pivot point and allows a tissue rotation of up to 180°. The donor site can often be closed primarily or be placed over muscle to allow skin grafting [10,11]. The perforators are usually located along the axis of the main vessels of the lower extremity [10,11,12,13,14,15,16,17]. Although complication rates have been shown to be higher than after microvascular transfer, pedicled flaps are utilized in patients, who may not qualify for microvascular tissue transfer due to age, comorbidities or other precluding conditions [11,18,19,20,21,22,23].

The free ALT flap and the pedicled propeller flap are among the most commonly used fasciocutaneous flaps for reconstruction of the lower extremity. Despite their morphological similarity, they exhibit distinct complication profiles. One potential contributing factor may be the torsion of the vascular pedicle in propeller flaps. The aim of this study was to analyze perioperative perfusion data of both flap types in order to identify potential predictors of complications. To assess perfusion characteristics in soft tissue, the laser Doppler spectrophotometry (LDSP) system can be used. With this non-invasive diagnostic tool, retrospective analysis of blood flow, hemoglobin concentration, and oxygen saturation was performed to identify and compare potential differences in perfusion between the two flap entities.

## 2. Methods

All adult patients (patients aged 18 years and older) who received soft tissue reconstruction of the lower extremity using a microvascular ALT or a pedicled propeller-type flap at the Clinic of Trauma, Hand, Plastic, and Reconstructive Surgery at the University Hospital of Würzburg between January 2017 and December 2021 were included in this retrospective study (*n* = 95). Patients who did not receive postoperative perfusion monitoring of the flap using the LDSP were excluded. The remaining patients were divided in two groups: pedicled flap (Propeller) vs. microvascular flap (ALT). Patient demographics, sex and age, as well as comorbidities were included. Perfusion monitoring data and perioperative data, including defect etiology, location, operating time, complications, surgical revisions, length of hospital stay were evaluated. Complications were defined as surgical complications, including partial and complete flap necrosis, epidermolysis, prolonged wound healing (defined as absence of completely healed wound edges by 2 weeks), and infection. Superficial epidermolyses, partial (not including necrosis of the wound margins) and complete flap necrosis were defined as complications caused by vascular impairment. The study was approved by the Institutional Review Board of our institution (Protocol ID:20210608 01, 18 October 2021 Julius Maximilians University, Wurzburg, Germany).

### 2.1. Perfusion Monitoring

Perfusion monitoring was performed using a laser Doppler spectrophotometry system (LDSP, O2C of LEA Medizintechnik GmbH (Gießen, Germany). The LDSP is a noninvasive diagnostic system with an external probe utilizing the two measurement methods. The spectrophotometry system detects the reflected light emitted by the probe (830 nm at 30 mW and 500–800 nm at 20 mW) and calculates the relative hemoglobin content (rHB in AU) as well as tissue oxygenation (SO_2_ in percent). As the emitted light is completely absorbed by vessels with diameters exceeding 100 µm, the light detected is reflected by the arterioles and venules of the dermal and subdermal plexus. Additionally, the laser Doppler method is used to detect the velocity of erythrocytes, which is then used to calculate the relative flow rate (Flow in AU).

The perfusion monitoring was performed at two different locations: the skin area above the perforator and the most distant tip of the flap representing the longest distance from the perforator. The region of the flap where the perforator could be identified by Doppler was marked. To ensure comparable measurements, values designated as *Perforator* were taken within a radius of approximately 1 cm around the detected perforator. The measurement designated as *Distal* was obtained at the point of the flap most distant from the perforator. In ALT flaps with a centrally located perforator, the distal measurement site corresponded to the distal end of the donor region. Measurements were obtained preoperatively at the planned and marked flap area the day before surgery, postoperatively on the day of surgery, the first, third, fifth, and seventh postoperative day. All measurements were obtained by the same surgeon with the LFX 33 probe (O2C of LEA Medizintechnik GmbH, Gießen, Germany). Measurements were carried out with the patient in a supine position 10 min prior to and during the measurements.

### 2.2. Perioperative Protocol

All patients underwent preoperative vascular imaging using CTA or angiography, and Doppler examination to identify and localize the perforator, as described by Yu et al. [24]. Furthermore, all patients received intraoperative anticoagulation with a bolus of 2000IE unfractioned heparin and postoperative anticoagulation with LMWH 40 mg 2× daily for 7 days. Postoperatively, patients were placed on bed rest with elevation of the involved extremity for five days, followed by a standardized mobilization protocol. Routine clinical flap monitoring consisting of visual inspection of skin color and capillary refill, as well as the hourly detection of the perforator with a handheld Doppler device during the first 48 h were carried out. Additionally, perfusion monitoring by LDSP was performed as described above.

### 2.3. Statistical Analysis

All data collected were anonymized and stored as Microsoft Excel documents and saved to an electronic clinical personal computer. All analyses were performed using Prism 10 (GraphPad Software, San Diego, CA, USA). Normality of continuous variables was assessed with the Shapiro–Wilk test due to the small sample size. For single comparisons (e.g., operative time), parametric or nonparametric tests were applied depending on distribution. For multiple comparisons, mixed-effects analysis was used with Geisser–Greenhouse correction, followed by Sidak’s post hoc test. Full models included column effect, row effect, and interaction terms. Categorical variables (e.g., smoking status) were analyzed using Fisher’s exact test. Correlation analyses were performed with Pearson’s or Spearman’s method, depending on data distribution. A *p*-value < 0.05 was considered statistically significant.

## 3. Results

### 3.1. Patient Demographics

Twenty-five patients were included in the analysis, of whom seven received soft tissue reconstruction with a pedicled Propeller flap and 18 with a microvascular ALT. In both groups male sex predominated (Propeller *n* = 6, 85.7%; ALT *n* = 11, 61.1%). Patient age in the Propeller and ALT groups was comparable at 62 ± 15.5 and 58 ± 14 years, respectively. The most common comorbidities were hypertension (Propeller *n* = 3, 42.9% and ALT *n* = 7, 38.8%) and arterial occlusive disease (Propeller *n* = 1, 14.3% and ALT *n* = 5, 27.8%). A third of the patients who received an ALT were smokers (Propeller *n* = 1, 14.3% and ALT *n* = 6, 33.3%). Trauma was the most common cause of defect in both the groups (Propeller *n* = 5, 71.4% and ALT *n* = 7, 38.9%), followed by chronic wounds (Propeller *n* = 1, 14.3% and ALT *n* = 5, 27.8%). The majority of the defects were located in the distal lower leg, ankle and foot (Propeller *n* = 5, 71.4% and ALT *n* = 15, 83.3%). Detailed information on patient demographics and comorbidities is shown in Table 1.

### 3.2. Perioperative Characteristics and Complications

Median operative time was 100 (IQR 70) minutes in the Propeller group and 235 (IQR 72.5) minutes in the ALT group. Negative pressure wound therapy was applied in almost all patients prior to secondary wound closure (Propeller *n* = 7, 100% and ALT *n* = 17, 94.4%). On average three surgical debridements were required prior definite closure in the Propeller and two in the ALT group. Bacterial cultures were obtained in all patients. Positive cultures were present in most patients prior to coverage (Propeller *n* = 6, 85.7% and ALT *n* = 15, 83.3%). The most common pathogen being Gram-positive cocci (Propeller *n* = 5, 71.4% and ALT *n* = 12, 66.7%). All patients with positive cultures received surgical debridements till negative cultures were obtained.

On average, the Propeller flaps were 94.3 cm^2^ in size. A perforator of the posterior tibial artery was used three times, the fibular artery twice and a perforator of the distal medial thigh twice. Five of the eighteen free ALT flaps were performed using two perforators, thirteen using one perforator. All perforators were eccentric and all microvascular anastomoses were performed end-to-side.

More than half of patients experienced surgical complications (Propeller *n* = 4, 57.1% and ALT *n* = 12, 66.6%). Delayed Wound healing was the most common complication (Propeller *n* = 3, 42.9% and ALT *n* = 8, 44.4%), followed by partial flap necrosis (Propeller *n* = 1, 14.3% and ALT *n* = 2, 11.1%). Complete flap loss occurred only in one patient in the ALT group. Surgical revision was needed in Propeller *n* = 2, 28.6% and ALT *n* = 6, 33.3%). Detailed information on perioperative characteristics and complications is shown in Table 2.

### 3.3. Perfusion Monitoring

**Figure 1:**
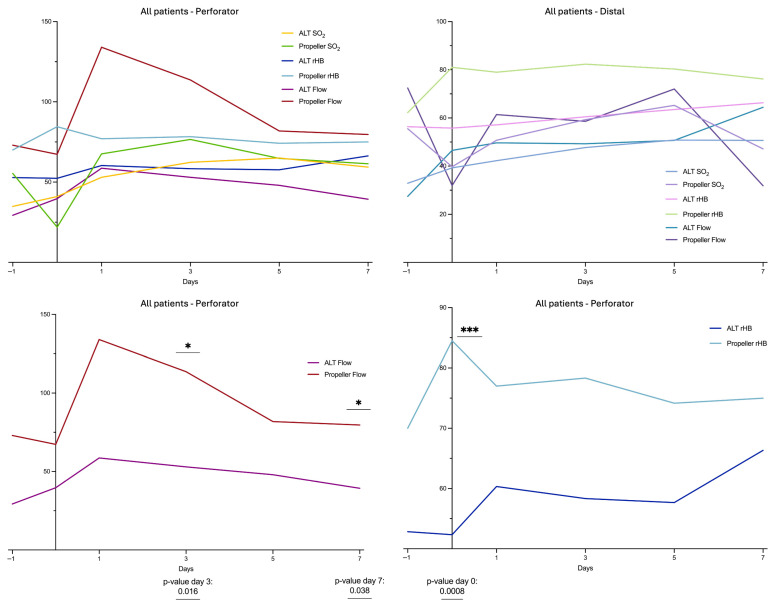
LDSP data of the Propeller and ALT group at perforator and distal flap site. Measurement quantities: relative Flow (in AU), tissue oxygenation (SO_2_ in %), relative hemoglobin content (rHb in AU); monitoring points of time: d−1: preoperative, d0: postoperative day of surgery, d1: first day postoperative, d3: third day postoperative, d5: fifth day postoperative, d7: seventh day postoperative. “*” indicates the significant difference between the Flow of the ALT group and the Flow of the Propeller group; see also the *p*-values listed below. “***” indicates the significant difference between the rHB of the ALT group and the rHB of the Propeller group; see also the *p*-values listed below.

Postoperative LDSP monitoring revealed noticeable differences between Propeller and ALT flaps. The Propeller group showed an increase in mean Flow at the site of the perforator of the flap with a peak at first postoperative day (mean Flow d1 124 ± 54 AU). In contrast, the ALT group showed a consistent course of the mean Flow over the perforator site as well as the distal tip area postoperatively. At the perforator site, significantly higher flow values were observed in the propeller group on postoperative days 3 and 7. Furthermore, an increase in mean rHB was observed in the Propeller group, which remained detectable in all measurements in the first week postoperatively (mean rHB d-1: Perforator 65 ± 8.5 AU; Distal 62.2 ± 6.3 AU; Mean rHB d0–d7: Perforator 77.8 ± 9.1 AU; Distal 78.3 ± 8.1 AU). Additionally, at day 0, rHB at the perforator site was significantly higher in the propeller group compared to the ALT group (Figure 1).

### 3.4. Comparison of Subgroups in Propeller Flaps

**Figure 2:**
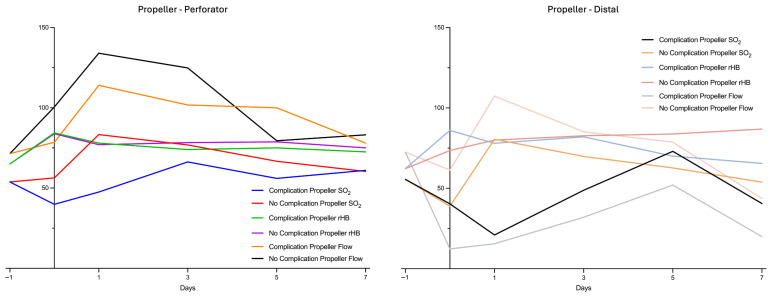
LDSP data of the subgroups (complication-free cases and cases with complications) of the Propeller group at perforator and distal flap site. Measurement quantities: relative Flow (in AU), tissue oxygenation (SO_2_ in %), relative hemoglobin content (rHb in AU); monitoring points of time: d−1: preoperative, d0: postoperative day of surgery, d1: first day postoperative, d3: third day postoperative, d5: fifth day postoperative, d7: seventh day postoperative.

Patients with postoperative complications after propeller flap surgery showed differences in perfusion patterns compared to those without postoperative complications. The subgroup with complications (*n* = 4) showed an immediate and pronounced decrease in mean Flow and tissue oxygenation (SO_2_) at the distal tip area the day of surgery (Flow 12, 3 ± 4.7 AU; SO_2_ 40, 3 ± 34.1%) and the first postoperative day compared to preoperative measurements (Flow 72.4 ± 26.3 AU; SO_2_ 55.6 ± 17.8%). Meanwhile, at the perforator site the Flow increased with a peak on the first postoperative day (Flow Perforator d1 114 ± 74 AU) compared to preoperative measurements (Flow 71.4 ± 7.7 AU). In contrast, in the subgroup without complications, there was a slight decrease in Flow and SO_2_ at the distal tip area directly postoperatively (Flow 61.3 ± 22 AU; SO_2_ 38.8 ± 2.1%) with a quick increase the day after surgery (Flow 107.3 ± 25.7 AU; SO_2_ 83.3 ± 6.3%) compared to preoperative measurements (72.4 ± 26.3 AU; SO_2_ 55.6 ± 17.8%). At the perforator site, there was a pronounced increase in Flow postoperatively with a peak on the first postoperative day (Flow 134 ± 12.3 AU) compared to preoperative measurements (Flow 71.4 ± 7.7 AU) (Figure 2).

### 3.5. Comparison of Subgroups in ALT Flaps

**Figure 3:**
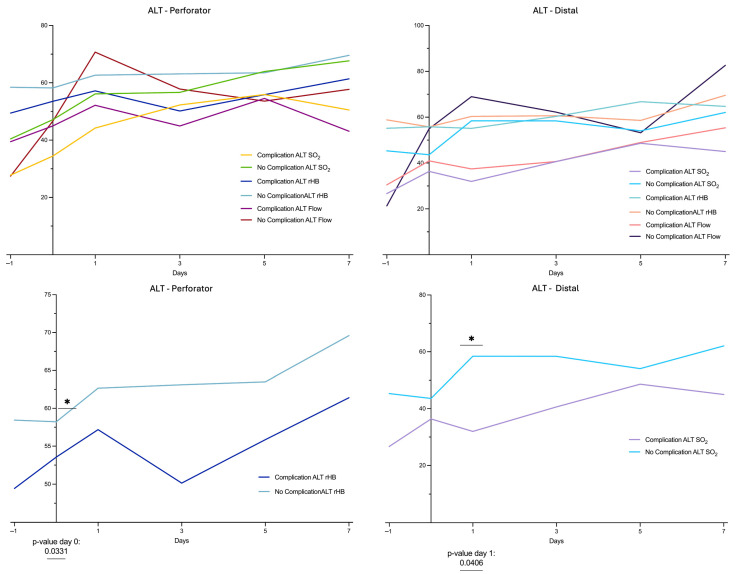
LDSP data the subgroups (complication-free cases, cases with complications) of the ALT group at perforator and distal flap site. Measurement quantities: relative Flow (in AU), tissue oxygenation (SO_2_ in %), relative hemoglobin content (rHb in AU); monitoring points of time: d−1: preoperative, d0: postoperative day of surgery, d1: first day postoperative, d3: third day postoperative, d5: fifth day postoperative, d7: seventh day postoperative. “*” indicates the significant difference between the rHB and SO_2_ of the ALT group with Complications and with No Complications; see also the *p*-values listed below.

Flow increased postoperatively in the complication-free group (*n* = 6) with a peak on the first postoperative day (Perforator Flow 63.3 ± 6.9 AU; Distal Flow 75.2 ± 5.0 AU). The SO_2_ followed slowly without pronounced peaks over the study period and was significantly higher on postoperative day 1 in the subgroup without complications compared to those with complications in distal vessels. Additionally, rHB at the perforator site was significantly higher at day 0 in complication-free cases compared to cases with complications (Figure 3).

### 3.6. Comparison of Subgroups in Propeller vs. ALT Flaps

**Figure 4:**
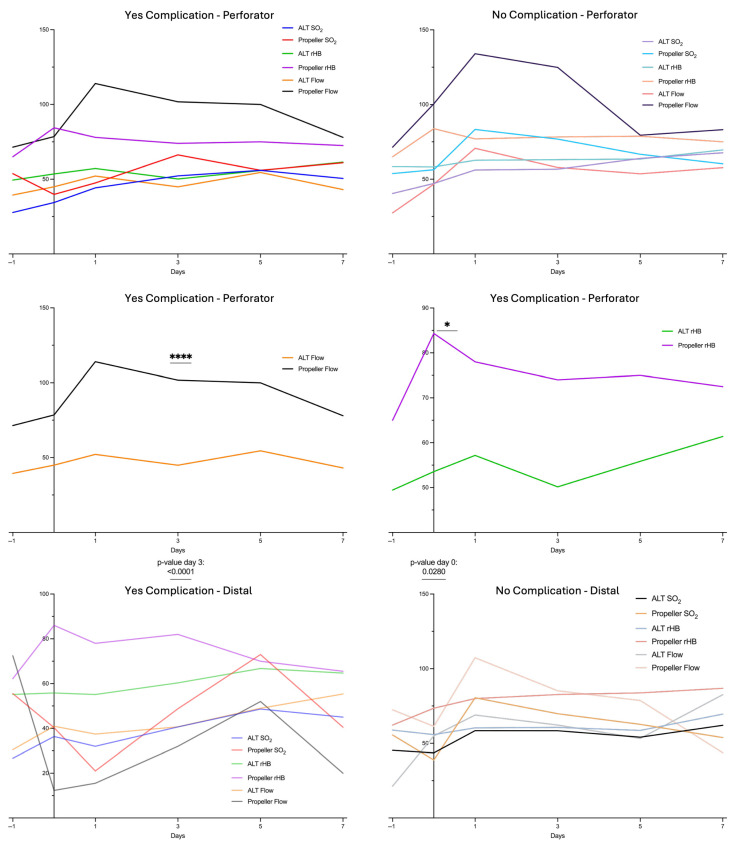
LDSP data of the subgroups (complication-free cases and cases with complication) of the Propeller group vs. ALT group at perforator and distal flap site. Measurement quantities: relative Flow (in AU), tissue oxygenation (SO_2_ in %), relative hemoglobin content (rHb in AU); monitoring points of time: d−1: preoperative, d0: postoperative day of surgery, d1: first day postoperative, d3: third day postoperative, d5: fifth day postoperative, d7: seventh day postoperative. “*” indicates the significant difference between the ALT rHb and Propeller rHb; “****“ indicates the significant difference between the Flow of the ALT group and the Flow of the Propeller group; see also the *p*-values listed below.

We further compared the LDSP data of the propeller subgroups with those of the ALT subgroups. A significantly higher Flow was observed on postoperative day 3 in Propeller flaps with complications compared to ALT flaps with complications at the perforator site (Propeller Flow 101.75 ± 2.25; ALT Flow 44.93 ± 14.13). In addition, rHB at the perforator site was significantly higher at day 0 in Propeller flaps with complications compared to ALT flaps with complications (Propeller rHB 84.3 ± 2.25; ALT rHB 53.56 ± 19.39) (Figure 4).

## 4. Discussion

Soft tissue reconstruction of the distal lower extremity is often necessary to avoid amputation of the extremity and allow patients to return to their pre-injured state.

Free tissue transfer has revolutionized extremity reconstruction and is considered the gold standard. The advent of perforator-based flaps has reignited surgical interest in local flaps. If an adequate perforator is present, a propeller flap represents a valid alternative to free tissue transfer in selected cases [11,12,25,26,27].

While partial flap loss is rare in free tissue transfer to the lower extremity, up to 33.3% of pedicled perforator flaps show prolonged healing at the distal tip [13,18,21,23,26]. The reason is not fully understood. It has been hypothesized that twisting of the vascular pedicle impedes mainly the venous outflow resulting in partial thrombosis. The results of this study confirm noticeable differences in the postoperative perfusion pattern between Propeller and microvascular flaps.

LDSP measurements demonstrated a marked postoperative increase in mean flow values at the perforator site of all propeller flaps, whereas the ALT group exhibited an unchanged perfusion pattern. This difference reached statistical significance on postoperative day 3 (Figure 1). Comparing the Propeller groups of cases with complications and without complications, a clear postoperative increase in blood flow of the cases without complications can be seen over the perforator. This increase can already be seen on the day of surgery and peaks on the first postoperative day. Even there is no significant difference, in the cases with complications of Propeller flaps, the increase in blood flow is much less pronounced and appears to set in much later. Additionally, delayed hyperperfusion in cases with complications appears to result in a marked decrease in blood flow in the distal vessels. A similar trend was observed for SO_2_, albeit with a temporal delay (Figure 2). The comparable groups of ALT flaps show a similar but much less pronounced Flow pattern (Figure 3).

However, the pathophysiological causes differ. A major reduction in blood flow of free ALTs is usually caused by a disruption of the inflow. Technical problems of the anastomosis in particular, but also thrombosis of the pedicle vessels are common causes [28]. In propeller flaps, reduced perfusion is mainly caused by twisting the pedicle. This is accompanied not only by an inflow disorder but also by an outflow disorder. As twisting of the vessel decreases the intraluminal diameter, the blood flow volume decreases exponentially (Hagen-Poiseuille Equation). The effect is aggravated in veins due to lower intraluminal blood pressure and thinner vessel walls [25]. The perfusion of the distal flap requires perfusion of the adjacent angiosome through either direct or indirect linking vessels [14]. When flow volume is already low in the first angiosome, passage to the next angiosome may be further impaired, as demonstrated by the marked reduction in blood flow at the distal tip in complicated cases within the propeller group. If perfusion, and consequently oxygen delivery, is critically reduced, hypoxic tissue damage will inevitably occur. Studies such as that by Bigdeli et al., which identified defect sizes greater than 100 cm^2^ as a significant predictor of major complications in reconstructions using propeller flaps, provide clinical support for this proposed pathomechanism [29].

Moreover, the Propeller flap group demonstrated a postoperative increase in rHb (Figure 1). The increase at the perforator site was found to be significant immediately after surgery (day 0) in comparison with the ALT group. Similarly, when comparing cases with complications to those without complications in both the Propeller and ALT groups, rHB was significantly higher in the propeller group with complications, already evident immediately postoperatively at day 0 (Figure 4). This might be explained by the compromised venous outflow due to twisting of the commitant veins of the pedicle and interruption of the venous outflow via the subdermal plexus. This causes a centralization to one or two commitant veins (relative outflow obstruction). Additionally, with the reduction in the arterial inflow after twisting the pedicle, the blood flow in the adjacent angiosome decreases. The venous outflow depends on a sufficient blood flow and pressure, otherwise resulting in a stasis. In line with our findings, Mitsutoshi et al. reported venous congestion as the most frequent early complication in propeller flaps, occurring in 72% of cases, and noted that it was significantly more often compared to free flaps [30]. Other studies have reported venous congestion rates ranging from 8 to 17% [13,31]. A delayed procedure as described by Chaput et al. might be a possible preventive surgical technique in critical patients [32].

Comparison between ALT flaps with and without complications also revealed significant differences in rHB and SO_2_. In the group with complications, rHB and SO_2_ were significantly lower postoperatively. Flow was also markedly lower, particularly at the distal flap region in cases with complications, although this difference did not reach statistical significance. The alterations observed are most consistent with an impaired arterial inflow, subsequently leading to ischemic changes. Most vascular-induced complications of free flaps occur within the first 24 h and can be resolved by early revision surgery [32]. Prompt surgical revision in cases of vascular impairment is considered crucial for outcome of free vascular tissue transfer [33,34,35,36].

The comparison of cases with complications to those without complications in both the Propeller and ALT groups revealed a significant higher Flow and rHB in the Propeller group at the perforator site. The significant divergence of these two parameters is further accentuated by the propensity of propeller flaps toward venous congestion, in contrast to the signs of inflow impairment observed in free flap reconstruction.

This study shows different perfusion patterns in different flap entities that can be visualized using the LDSP. However, there are limitations to this study. The retrospective structure of the study implies confounders and bias. In addition, there is a bias due to inclusion criteria, as not all free ALTs and Propeller flaps performed in 2017–2022 were included as vascular monitoring was not performed in all cases. An additional source of bias may be the necessity of meticulous surgical dissection of the perforator during preparation of the propeller flap. Perfusion may be impaired due to torsion and traction if dissection is not performed with sufficient care. It should also be noted that the LDSP represents only a locally limited tissue measurement. The assessment of larger areas to visualize perfusion changes is currently not possible with this device. False positive or negative measurements not representing the general perfusion of the monitoring area are possible. Moreover, the acquisition of measurements was performed at defined intervals during the first postoperative week. Continuous measurements would be preferable to detect vascular impairment. Furthermore, the LDSP perfusion monitoring is operator-dependent with no validated cut-off values and a good knowledge of LDSP and continuous monitoring is necessary to recognize vascular impairment. This study is also limited by the small group size, which is inherent to the infrequent utilization of the pedicled propeller flap method. Therefore, we used a full mixed-effects model including column, row, and interaction effects to analyze repeated measures data. While normality assumptions were not fully met due to small sample size, we applied the Geisser–Greenhouse correction to account for violations of sphericity and Sidak’s post hoc tests for multiple comparisons. Importantly, mixed-effects modeling was selected because it permits inclusion of missing values and testing of interaction terms, which cannot be accommodated by standard non-parametric approaches such as Friedman tests. Although alternative methods (e.g., rank-based or permutation approaches) may offer robustness for non-normal data, they are less practical in this setting. Therefore, results should be interpreted with caution, acknowledging that mixed-effects models provide the best available framework for evaluating time-course changes and group per time interactions in our dataset.

It should also be noted that flap size could not be analyzed as a variable, as consistent documentation of ALT flap dimensions was lacking. Very large free ALT flaps, up to 35 × 25 cm, have been reported in the literature [37] and are typically reserved for the reconstruction of extensive defects, with intraoperative angiographic verification recommended in such cases. While larger ALT flaps may be more prone to perfusion-related complications, partial necroses rarely compromise overall flap survival and can often be managed by revision surgery. Due to the limited sample size and incomplete data, no reliable correlation between flap size and perfusion could be established, highlighting the need for future prospective studies to address this issue.

## 5. Conclusions

In conclusion, this exploratory analysis provides preliminary insights into potential differences in perfusion patterns between two perforator-based flap types, both over time and within the flap itself. Propeller flaps may be more prone to congestion, with a tendency toward elevated rHB and delayed hyperperfusion, whereas ALT flaps may show a more consistent profile with inflow dependence, reflected in comparatively lower rHB and SO_2_. These observations are hypothesis-generating and should be viewed as tentative given the retrospective design and limited sample size. Future prospective studies are needed to validate and further elucidate these potential patterns and their clinical implications.

## Figures and Tables

**Table 1:** Patient Demographics and comorbidities. Reported as *n* (%), unless otherwise stated.

Characteristics	Propeller (*n* = 7)	ALT (*n* = 18)	*p*-Value
**Demographics**			
Male sex (*n*)	6 (85.7)	11 (61.1)	0.36
Female sex (*n*)	1 (14.3)	7 (38.9)	0.36
Age, median years (IQR)	62 (25)	58 (18)	0.95
**Comorbidities**			
Hypertension	3 (42.9)	7 (38.9)	>0.99
Diabetes	2 (28.6)	1 (5.56)	0.18
Arterial occlusive disease	1 (14.3)	5 (27.8)	0.64
Nicotine	1 (14.3)	6 (33.3)	0.63
**Defect Cause**			
Trauma	5 (71.4)	7 (38.9)	0.20
Cancer	1 (14.3)	1 (5.56)	0.49
Chronic Wound	1 (14.3)	5 (27.8)	0.64
Infection	0 (0.00)	2 (11.1)	>0.99
Wound healing disorder	0 (0.00)	2 (11.1)	>0.99
Autoaggression	0 (0.00)	1 (5.56)	>0.99

**Table 2:** Perioperative Characteristics and Complications. Reported as n (%), unless otherwise stated.

Characteristics	Propeller (*n* = 7)	ALT (*n* = 18)	*p*-Value
Operative time, median minutes (IQR)	100 (70)	235 (72.5)	**<0.0001**
Length of hospital stay, median days (IQR)	28 (9)	22 (17)	0.58
Debridement prior coverage	3 (42.9)	2 (11.1)	0.11
Negative pressure wound therapy	7 (100)	17 (94.4)	>0.99
Microbial contamination prior to coverage	6 (85.7)	15 (83.3)	>0.99
**Complications**			
Patients (*n*)	4 (57.1)	12 (66.7)	0.67
Prolonged wound healing	3 (42.9)	8 (44.4)	>0.99
Superficial incisional infection	0 (0.00)	2 (11.1)	>0.99
Epidermolysis	2 (28.6)	1 (5.56)	0.18
Partial flap necrosis	1 (14.3)	2 (11.1)	>0.99
Complete flap necrosis	0 (0.00)	1 (5.5)	>0.99
Flap survival	7 (100)	17 (94.4)	>0.99
Reoperation	2 (28.6)	6 (33.3)	>0.99

## Data Availability

The original contributions presented in this study are included in the article. Further inquiries can be directed to the corresponding author.
